# What I Wish I Had Known: Examining Parent Accounts of Managing the Health of Their Child With Intellectual Disability

**DOI:** 10.1111/hex.70138

**Published:** 2025-01-09

**Authors:** Thom Nevill, Jessica Keely, Rachel Skoss, Rachel Collins, Katherine Langdon, Jaquie Mills, Jenny Downs

**Affiliations:** ^1^ The Kids Research Institute Australia, Centre for Child Health Research University of Western Australia Perth Australia; ^2^ Institute for Health Research University of Notre Dame Perth Australia; ^3^ School of Population and Global Health University of Western Australia Perth Australia; ^4^ CAHS Transition Service Perth Children's Hospital Perth Australia; ^5^ Kids Rehab Perth Children's Hospital Perth Australia; ^6^ Microboards Australia Perth Australia; ^7^ Curtin School for Allied Health Curtin University Perth Australia

**Keywords:** chronic conditions, health literacy, health management, Intellectual disability, parent perspective, young people

## Abstract

**Background:**

Appropriate support for the health of children with an intellectual disability by parents and healthcare professionals is pivotal, given the high risk of chronic conditions. However, there is limited research that has collected important insights from parents on their learnings for supporting their child's evolving healthcare needs.

**Aim:**

This study focuses on parents' experiences and learnings from managing and supporting the health of their child with intellectual disability. It aims to understand what parents wish they had known earlier, the essential knowledge and skills they needed to manage their child's health.

**Method:**

A qualitative study was carried out using semi‐structured interviews with 21 parents of adolescents and young people with intellectual disability. The children had chronic health conditions that fell into six health domains, including (1) difficulties with movement and physical activity, (2) epilepsy, (3) dental care, (4) respiratory health and infection, (5) behaviour, mental health or sleep and (6) gastrointestinal health.

**Results:**

Thematic analysis yielded five themes: (1) optimising mutual engagement between healthcare professionals and families; (2) planning and practising effective healthcare; (3) having the right information at the right time; (4) finding the support that was needed and (5) navigating healthcare and disability systems. Over time, parents developed specific skills and knowledge for managing their child's health effectively. Some parents expressed regret for not seeking support and information about their child's health conditions earlier. Parents described how mutual engagement between healthcare professionals and parents optimised the management of their child's healthcare.

**Conclusion:**

The study found that managing the health of a child with intellectual disability is complex. The themes were consistent across health comorbidities, indicating important common experiences. The themes aligned with conceptualisations of health literacy, suggesting that improving health literacy skills can help parents better manage their children's health conditions.

**Patient or Public Contribution:**

We developed the project in consultation with members of the public who have lived experience of parenting a child with intellectual disability. They commented on the study aims, interview schedule, participant recruitment and provided feedback on the analysis and discussion.

## 1 Introduction

Children with intellectual disability are at increased risk of chronic physical, neurological and behavioural conditions and have a greater need for healthcare services than is usually required by children without intellectual disability [1, 2]. The main comorbidities experienced by people with intellectual disabilities can be grouped into six broad health domains including (1) challenges with movement and physical activity, (2) epilepsy, (3) dental care, (4) respiratory health, (5) gastrointestinal health and (6) behaviour, mental health and sleeping disturbances [3, 4, 5]. Certain comorbidities, such as epilepsy and mobility challenges, can be influenced by the cause and severity of the intellectual disability [6]. Other comorbidities, for example, sleep disturbances and mental health problems, are common regardless of the underlying cause of intellectual disability [3, 7]. Health problems and high rates of healthcare service utilisation persist into adulthood [8].

It is pivotal to appropriately support the health and well‐being of children with intellectual disability, given the high risk of chronic health conditions. Primary caregivers, most typically mothers, play a crucial role in managing the healthcare of all children, including those with intellectual disability [9]. Parent experiences supporting the health of children with disabilities are complex, involving daily health management routines, the utilisation of multiple healthcare services, and interactions with a variety of healthcare professionals [10, 11]. Throughout their healthcare journeys, parents of children with intellectual disability can face challenges linked to accessing services, managing the transition to adult services and difficulties getting supports [12]. Despite these challenges, most parents acquire the health knowledge and skills to effectively manage their children's health over time [13]. Much of the literature exploring how parents support the health of children with intellectual disability has focused on their experiences in hospitals [9, 14, 15], where the importance of parental advocacy for children to receive appropriate assessments and treatments is highlighted [16, 17]. Beyond hospital care, reviews describe how to support parents in managing stress [18], unscheduled healthcare for children with intellectual disabilities [19] and parents' experiences of general practice support [20].

To our knowledge, no study has collected salient insights from parents on their learnings from supporting their child's evolving healthcare needs with increasing age, focusing on what they wish they had known. Identifying what parents wish they had known earlier in their journeys is crucial for clinical practice and understanding how to support parents during different phases of their child's development. In this study, we aimed to (1) describe what parents wish they had known and done earlier in their child's health journey, (2) examine the core knowledge and skills parents felt were necessary to effectively manage their child's health across home and medical settings, and (3) understand parents' perspectives on how to optimise interactions with healthcare professionals.

## 2 Methods

The University of Western Australia Human Research Ethics Committee provided ethical approval for this study (2021/ET000902). Informed consent was provided before interviews began. The researchers who completed the interviews and analysis have family members with disability and experience in disability research.

### 2.1 Recruitment and Sample

Participants were recruited from databases housed at The Kids Research Institute Australia. They were eligible if they had a child aged 9–25 years with intellectual disability and one or more chronic health conditions. We included children aged 9 or older to capture lessons and reflections over early and early middle childhood, a period of time that would enable accumulation of experiences. Recruitment strategies aimed to capture participants with children across a range of settings (metropolitan and rural), ages and with different chronic health conditions. Twenty‐eight parents were contacted and invited to take part in the study via phone and/or email. One parent declined to participate and six withdrew before being interviewed.

It was predicted that a sample size of approximately 20 participants would adequately achieve information power [21]. Information power identifies an appropriate sample size based on the brevity of the research aims and focus (broader – larger samples), methods undertaken, and data quality (rich and deep data – smaller samples). For this study, the broad aims and focus (healthcare experience) were considered in relation to the opportunity to collect rich quality data (in‐depth interviews with parents with extensive experience).

The final sample consisted of 21 parents (20 mothers and 1 father). The children ranged from 9 to 25 years of age (median 17 years), slightly more than half (*n* = 11, 52.4%) identified as female. Most families lived in metropolitan areas (*n* = 16, rural *n* = 5). The children had a range of walking, communication and eating abilities. Thirteen (62%) had been diagnosed with epilepsy. Fourteen had seen more than four types of medical specialists in the previous 6 months (Table 1).

**Table 1:** Description of the children and young people with intellectual disability (*n* = 21).

	*N*
**Age**	
Child and adolescent (9–18 years)	16
Young adult ( > 18 years)	5
**Gender**	
Female	11
Male	9
Other	1
**Location**	
Metropolitan	16
Rural	5
**Primary diagnostic groups (in addition to intellectual disability)**	
Down syndrome^a^	5
Cerebral palsy	3
Autism spectrum disorder	3
Other^b^	10
**Number of types of medical specialists seen in past 6 months**	
1–4	6
5–9	11
10+	3
Unknown	1
**Mobility**	
Can walk independently	10
Can walk with assistance	4
Cannot walk	7
**Communication**	
Speaks well – understood by all	4
Uses speech for communicating but some difficulty understanding	10
Uses speech and assistive technology to communicate (e.g., AAC devices)	3
Uses nonverbal communication primarily with the aid of some assistive technology	4
**Eating and swallowing**	
Eats food orally	14
Eats food orally, but some difficulty with swallowing	1
Eats some food orally and some through a gastrostomy tube	2
Has food via a gastrostomy/jejunostomy/nasogastric tube	4
**Diagnosis of epilepsy**	
Yes	13
**Treatment for breathing problems (over the last 12 months)**	
Antibiotics	5
Hospitalisation	1
**Challenging behaviours**	
Yes	8

^a^ Two with comorbid autism spectrum disorder.

^b^ Includes genetic syndrome (*n* = 5), no formal diagnosis (*n* = 3), and epilepsy syndrome (*n* = 2).

### 2.2 Materials

A brief questionnaire was administered before commencing interviews. Questions enquired about the child's current communication, functional abilities, and health needs (e.g., ability to walk, how the child communicates pain, oral or enteral feeding). Six sets of questions were designed to explore the experiences and learnings of parents of children with intellectual disability and chronic health conditions across different domains of health that were important to them, including (1) movement and physical activity, (2) epilepsy, (3) dental care, (4) respiratory health and infection, (5) behaviour, mental health, and sleep and (6) gastrointestinal health (see Appendix A). Interview guides asked about domain experience generally (e.g., ‘Can you tell me a bit about your child's movement?’), experiences with diagnosis, ongoing management of conditions (e.g., access to equipment and medication management), lessons learnt (e.g., ‘What has been the most important thing you've learned about the day‐to‐day management of the condition?’) and interactions with healthcare professionals (e.g., supportiveness). Open‐ended questions were used to explore experiences throughout the child's health journey while probing questions sought to elicit further explanation.

### 2.3 Procedure

During recruitment, participants indicated which of the domains they wished to discuss in detail in their interview. The interview guides were administered as follows: behaviour, mental health and sleep (*n* = 11), dental care (*n* = 8), movement and physical activity (*n* = 7), gastrointestinal health (*n* = 6), respiratory health and infection (*n* = 6) and epilepsy (*n* = 5). Most participants indicated that they had experience supporting their child's health across multiple health domains and several domains were often discussed.

Interviews were audio‐recorded and conducted via video call by J.K. Additionally, field notes were taken throughout the interviews. Interviews were transcribed verbatim and checked for accuracy by J.K. and T.N.

### 2.4 Thematic Analysis

Thematic analysis was conducted by two researchers following the six‐step process outlined by Clarke and Braun [22] using NVivo (10th ed., QSE International Pty Ltd., Burlington, MA). These stages include (1) familiarising yourself with the data, (2) generating initial codes, (3) searching for themes, (4) reviewing themes, (5) defining and naming themes and (6) producing the report. During the first stage, two members of the research team familiarised themselves with the data by reading and rereading transcripts closely. In Stage 2, both researchers generated an initial list of codes inductively. This was achieved by two researchers independently coding the first three interviews and then meeting to discuss and compare their coding, consolidating any minor differences. Both researchers then coded the first half of the transcripts and met to review the coding scheme. After establishing that coding was consistent between the researchers one researcher coded the remaining interviews.

For Stage 3, related codes were grouped into themes, named and defined. Several codes were consolidated to form themes that captured an underlying concept identified in the data. For example, codes including ‘good healthcare providers are empathetic and sympathetic’, ‘and ‘valuing relationships with healthcare providers’ were merged to form one theme ‘optimising mutual engagement between healthcare professionals and families’. Throughout this process, the researchers became aware that the identified themes related to nine domains included in the model of health literacy outlined in Batterham et al. [23]:

1. Feeling understood and supported by healthcare providers.
2. Having sufficient information to manage my health.
3. Actively managing my health.
4. Social support for health.
5. Appraisal of health information.
6. Ability to actively engage with healthcare providers.
7. Navigating the healthcare system.
8. Ability to find good health information.
9. Understanding health information well enough to know what to do.

Accordingly, the names of certain sub‐themes were re‐named, where appropriate, in alignment with the health literacy model. In Stage 4, the research team collaboratively reviewed the themes to ensure they were coherent, captured distinct concepts and comprehensively captured the participant's experiences. Both researchers developed the names and definitions of the themes and then evaluated and refined them in discussion with the research team (Stage 5), after which a report was produced (Stage 6).

An audit trail was created during the coding process to ensure the analysis was rigorous and systematic. This was achieved by saving copies of the project in NVivo at each analysis stage and recording any key changes made to the coding scheme through journaling.

## 3 Results

Five overarching themes emerged from the data: (1) optimising mutual engagement between healthcare professionals and families; (2) planning and practising effective healthcare; (3) having the right information at the right time; (4) finding the support you need and (5) navigating healthcare and disability support systems. These themes captured the complex mix of factors that parents felt were critical to effectively manage and support their child's health. They also encapsulated what they wish they had known or done earlier, and ways that healthcare providers can support families. These themes consistently emerged across all health domains; however, differences were observed in the salience of certain themes based on a child's specific health condition and diagnosis.

### 3.1 Theme 1: Optimising Mutual Engagement Between Families and Healthcare Professionals

Parents emphasised the importance of their interactions and relationships with healthcare professionals in managing their child's health. Optimising engagement between families and healthcare professionals was discussed as a two‐way process that hinged on both parties contributing beneficial skills and traits. This theme has two parts: (1) parent factors and (2) healthcare professional factors, to consider how each party can work to optimise interaction outcomes.

#### 3.1.1 Parent Factors

Participants described specific skills and approaches that helped them to engage effectively with healthcare professionals. First, participants identified that it was important to feel comfortable asking healthcare professionals questions. Several participants shared that they initially felt intimidated when asking questions and in hindsight, wished they had overcome this concern sooner. Asking questions was perceived as important to obtaining the information from healthcare professionals that they needed to make informed healthcare decisions. For example, when providing advice for other parents, one mother (P1):

I think you need to not be afraid to ask questions. It's that old adage, no question is a stupid question.

Second, parents reported that advocating for their child's needs was important in healthcare settings because it helped ensure that parent knowledge contributed to clinical decision‐making. Participants outlined that the need to advocate for their child was particularly salient in hospital settings due to their child's communication difficulties, particularly around experiences of pain and discomfort. Describing her experience with her non‐verbal son, one mother (P2) illustrated the importance of being a strong advocate when your child doesn't outwardly express pain:

You can never stop advocating for your child. … even with the most willing and wonderful doctors and health professionals, you just have to keep at it … When your child's putting on a brave face, you know your child … (so) you need to tell the doctor that.

In this example, the parent acknowledges that being an advocate is challenging and articulates how they use their knowledge of their child to advocate for their needs.

Participants described how their advocacy skills developed over time as they became more experienced. Describing how she became more confident in her advocacy one mother (P3) said

In the early days, you're very much listening to the medical professionals because they know more than you. As time goes on and you experience more and you learn more, I think you just naturally become stronger in your advocacy.

While participants emphasised the need for assertive advocacy, they also recognised that building strong relationships with healthcare professionals through open dialogue and mutual respect was a fundamental platform for effective engagement.

#### 3.1.2 Healthcare Professional Factors

Alongside parent factors, participants outlined how healthcare professionals could optimise their engagement with families. Participants reported that supportive healthcare professionals were empathetic, good listeners and valued parent perspectives. Healthcare professionals possessing these qualities enabled collaborative and empowering relationships with participants, positively impacting child health management. For example, one mother (P4) described her relationship with her daughter's paediatrician as being based on honesty, trust and curiosity:

He was a real communicator. You just knew when you walked into his office that he was there for my daughter. You felt confident that he understood what was going on and felt confident in taking his advice. He would always listen. We would discuss backwards and forwards what we thought.

In contrast, participants provided examples of healthcare professionals whom they perceived had not listened to them and showed little empathy. A mother (P5) outlined the importance of healthcare professionals being empathetic when she described how she was informed of her son's diagnosis:

…. Even when they rang me to tell me that your child has [diagnosis], I was driving in a car, and they didn't even say, are you with someone or are you alone.

It was also considered crucial that healthcare professionals engaged directly with the child. Some participants described healthcare professionals who included the child in discussions, communicated clearly, and created an environment for them to be comfortable. For example, one mother (P6) described the steps that healthcare professionals at a dental clinic took to ensure her son was comfortable and prepared for his medical procedure:

She actually engaged with my son. She addresses him, the staff do as well, they address him by his name. They try and make eye contact, they welcome him, they reassure him. And often they darken the room, so it was a bit more soothing.

In this example, health outcomes were improved by communicating clearly and personably and considering the child's emotional and sensory needs. In addition, participants emphasised that supportive healthcare professionals were willing to work collaboratively with professionals from other specialties. Healthcare professionals working together in a team was viewed as critical to effectively coordinating care, especially when a child has multiple health conditions. As one example, a mother (P7) explained how she valued the ‘open dialogue’ and ‘regular communication’ between different medical and allied health specialists related to her daughter's respiratory health.

### 3.2 Theme 2: Planning and Practising Effective Healthcare

This theme describes how effective management of the health of children with intellectual disability is an evolving and ongoing process. Effective management is supported by parents' understanding of their child's health and involves different strategies. This theme is organised into four sub‐themes: (1) Ongoing and iterative process of optimising health management, (2) Understanding signs and symptoms of changing health status, (3) Importance of preventive care, (4) Building child's involvement in the management of their healthcare. These sub‐themes capture different aspects of the approaches used by parents to manage their child's health.

#### 3.2.1 Ongoing and Iterative Process of Optimising Health Management

Participants emphasised that it can be an ongoing process to identify what causes a health condition, often iterative as potential causes are ruled out. Similarly, determining how to manage the condition effectively can take time. This was predominantly discussed by parents whose children had epilepsy or a genetic condition. Due to the very complex nature of their children's conditions, these participants faced unique challenges in finding the correct diagnosis for their child and then the most suitable treatment that took time. One mother (P13) described how finding the correct diagnosis and identifying the best treatment for her son took several years:

It started when he was 2 years and 3 months and he's 18 now…. In the beginning, they just called it a generalised seizure disorder. As time went on, they thought he had Lennox‐Gastaut syndrome…Unfortunately, there hasn't been (one way of resolving the seizures), he's on about five different drugs now … he still has seizures, but certainly a lot better now.

Parents whose children had less complex health conditions also reported finding effective approaches through trial and error with their clinician. For example, several participants described how they tried an array of treatments, including melatonin, different medications and light therapy, before settling on an effective approach to managing their child's sleeping difficulties. For example, one mother (P10) described the process it took to get her two sons to sleep:

It's just been a journey, working with a team of different specialists to get us to this point where they both now sleep beautifully through the night.

These parents' experiences illustrate that it is important to understand that determining the most effective approach to managing health conditions is not always straightforward. This was especially pertinent for parents whose children have complex conditions.

#### 3.2.2 Understanding Signs and Symptoms of Changing Health Status

Parents reported they felt better equipped to manage their child's healthcare when they were able to recognise the sometimes subtle signs and symptoms that indicated onset of illness. This sub‐theme was most prevalent among parents whose children experienced communication difficulties. Parents often commented that their children experienced difficulty expressing pain, noting it took time to notice certain changes in their child's behaviour or body language that indicated pain. For example, a mother of a son with Fragile X syndrome (P10) explained how she became more aware of how her son communicated pain after he had a serious urinary tract infection:

It just didn't occur to me at all that would've been why all his behaviours were escalating. He kept telling me he needed to go to the toilet. He was just so anxious. There was no communication from him at all. The aha moment for me was piecing together more of his body language, the signs that I'd missed. I'm probably just a bit more clued in (now) to piecing together.

Recognising signs that indicated a change in their child's health assisted parents to more confidently advocate for their child in healthcare settings. For example, a mother (P5) explained that understanding the specific ways her daughter with Down syndrome and autism exhibited pain meant she was better prepared to advocate for her to receive appropriate healthcare assessment and management:

I usually explain to people that for her to exhibit any pain means that she's in a lot of pain because she doesn't outwardly exhibit pain. I generally let people know in hospital settings [at the outset]. I analysed it myself at home, that's the reason we brought her here.

Being aware of the signs and symptoms that indicate a change in health status was also discussed as critical to the ongoing management of their children's healthcare, such as observing the impacts of medication.

#### 3.2.3 Importance of Preventive Care

Parents took proactive measures to optimise their children's healthcare by planning and practising regular preventive care. For example, parents of children with mobility challenges recommended promoting children's physical health by incorporating as much movement into their daily lives as possible. For example, a mother (P7) described how getting her non‐ambulant daughter on a walking frame twice a day helped promote different aspects of her physical health:

We put her in the standing frame twice a day. Even if it's just half an hour in the morning and half an hour at night, it's fantastic because being upright helps you breathe better, digest better, and develop denser bones.

Discussing respiratory health, one mother (P11) described how she adopted several measures to protect her son's immune system:

Trying to keep his immune system well‐supported with good nutrition and prophylactic antibiotics, but also vitamin and mineral supplementation where it's required and keeping a good eye on that barrel, the things that we put in place every day to manage his susceptibility.

Some participants reflected that they wished they had practised preventative care, including incorporating more physical activity in their children's day and introducing support for their child's mental health earlier and strongly encouraged other parents to be preventive in their approach, arguing that it would contribute to children's overall health.

### 3.3 Building Child Involvement in the Management of Their Healthcare

Some participants described the importance of increasing the involvement of children in managing their healthcare. This was seen as a significant step in empowering children to have more control and independence as they grow up. Participants increased their child's involvement in managing their health by preparing them to assume certain responsibilities, such as taking their medication. For example, one mother (P12) described as her daughter became older, she gave her more responsibility in the daily management of her epilepsy:

We've tried to make her responsible as well. So, she carries her emergency bag, which has her midazolam in and a flannel and contact numbers, which she wears whenever she goes out. Now she is more in charge of taking her medication.

### 3.4 Theme 3: Having the Right Information at the Right Time

Participants emphasised it was crucial to be informed about their child's health condition and recommended that other parents should be informed also. A variety of information sources were mentioned, such as the internet, other parents, advocacy organisations, and healthcare professionals. Other parents were viewed as particularly valuable sources of information. As one mother (P14) explained ‘*I didn't know that carers allowance existed until I talked to the parents at school’*.

Parents needed different types of information at different stages of their healthcare journey. Initially, after receiving a diagnosis, they required foundational information about the cause of the child's disability or health conditions, its impact, possible comorbidities and where they could find support. Such information was critical to participants developing an understanding of their child's health condition(s) and how to manage them. As their child grew older, participants needed new information to make informed decisions about managing their child's health, including what services are available for adults with intellectual disability as their child approached the transition from paediatric to adult services.

While participants described feeling capable of finding and then applying health information, some parents said that, at times, they wished they had accessed or been provided information earlier so they could be better prepared for the future. For example, a mother (P12) reflected on her information needs after her son was diagnosed with epilepsy:

We didn't receive advice [on] … how long it would last, whether she would have it for the rest of her life or until she became a teenager. What supports were down here (in rural town) that might help me as a parent to talk to somebody about?

We note that while participants recommended that parents should actively ask for information, healthcare professionals are not always able to provide answers to their questions because evidence may not be available.

### 3.5 Theme 4: Finding the Support You Need

The participants highlighted the importance of receiving support to manage their child's health and maintain their well‐being. Parents stressed the importance of understanding that they cannot do everything by themselves and that they need people they can go to for emotional, informational, and practical support. Several participants reflected that wished they had recognised the importance of finding these supports earlier.

Peer support was found to be particularly valuable because parents appreciated speaking with individuals who had similar or shared experiences, making them feel less lonely, and sometimes providing helpful advice. A mother (P7) highlighted the value of peer support when she described her experiences attending a playgroup organised by an epilepsy association:

It was the best because you could speak to mums who have kids, roughly the same age. They're young and you're in the same boat. You can compare notes sort of thing. And just being able to talk to someone who's in the same boat as you, just makes a world of difference.

The participants also shared how they established support networks including family members, healthcare professionals, friends, and members of support organisations. Some participants expressed a wish that they had recognised earlier that they did not need to manage all of their child's health by themselves. For example, explaining her main advice for others, one mother (P10) referred to the benefits of parents having a support network of professionals:

You've got to lean on your village and call up the experts who know how to deal with things and ask for help instead of trying to figure it out on your own. I did that for too long, and you can't do it on your own. You need a village. You need to call on your therapists, the experts, and your teachers.

Participants also discussed the need for parents to practice self‐care and often reflected they wished they had looked after their well‐being earlier in their journey. For example, one mother (P14) reflected:

I wish, probably I'd taken care of myself a bit better…. I don't even know if there was (support) for me, if it's offered to the mom or the dad that's living it day and night. So, my mental health probably suffered … through that journey.

Participants recommended that parents practice self‐care because it safeguards their mental health and gives them more energy to support their child's health.

### 3.6 Theme 5: Navigating Healthcare and Disability Systems

Participants described the complexity of navigating different processes and services within the healthcare and disability systems. The challenges participants experienced varied depending on their child's age. For example, participants with adolescent and adult children identified that transitioning from child to adult healthcare services was a particularly difficult process. Transitioning to adult healthcare posed challenges including finding new services and adjusting to a more fragmented and siloed healthcare system. Commenting on her daughter's transition into adult services, a mother (P15) described the core challenges she had grappled with:

We're transitioning into the adult world, with her being 18. … The transition team's great and all the letters are going through, but we've heard nothing from the adult world. A lot of the drugs that my daughter is on aren't PBS‐covered either. So, until we get picked up by the adult world and the lung team, there's a lot of drugs like tobramycin. That's nearly $500 a course.

Participants advised that it is important to commence planning for transition early and highlighted the benefits of having a competent transition team.

Parent experiences of navigating healthcare and disability support systems were also influenced by their child's specific healthcare needs. For instance, managing approval processes within the National Disability Scheme (NDIS) was a salient issue for participants whose children have a physical disability. Participants described their frustration with processes, especially wait times for vital equipment (e.g., wheelchair) and the extensive reporting procedures. One mother (P11) with a non‐ambulant daughter described her experience of accessing equipment through the NDIS:

It takes a long time, and it's always taken a long time. We were in the old system before NDIS and then we had a taste of WA NDIS. They all took and take a long time. Prescription takes a long time, and equipment arriving takes a long time. These are kids that are growing dynamically. They need to be supported quickly, and that doesn't happen.

Echoing the other key themes, participants shared how important it was to have health literacy skills such as knowledge of eligibility for services (Theme 3) and having a good support network to seek advice from (Theme 4) to navigate bureaucratic processes successfully.

## 4 Discussion

This study explored parent perspectives on managing the health of their children with intellectual disability and various accompanying health conditions. We aimed to understand what parents wish they had known or done earlier in their journeys, the essential skills and knowledge for managing a child's health, and their perspectives on optimising interactions with healthcare professionals. Participants discussed how they learnt from their experiences and recommended that other parents develop particular skills and knowledge to manage their children's health (e.g., find quality information, ask healthcare professionals questions, and access services). Five key themes were consistent across the various disability and health conditions, indicating important and common experiences and perspectives.

Parents reflected on different facets of what they wished they had known at the beginning of their child's health journey. Consistent with prior research, the results illustrate that managing the health of children with intellectual disability is complex, requiring ongoing management, planning, and interactions with a variety of healthcare professionals [9]. Parents often wished they had sought more social support and known more about specific aspects of their child's health condition, earlier in their child's healthcare journey. Future research should explore how to support parents best early in their child's health journey and develop interventions to encourage social engagement. While some participants expressed a desire to have handled certain things differently, it was through their experiences that they acquired the skills and knowledge necessary to manage their children's health effectively.

**Figure 1:**
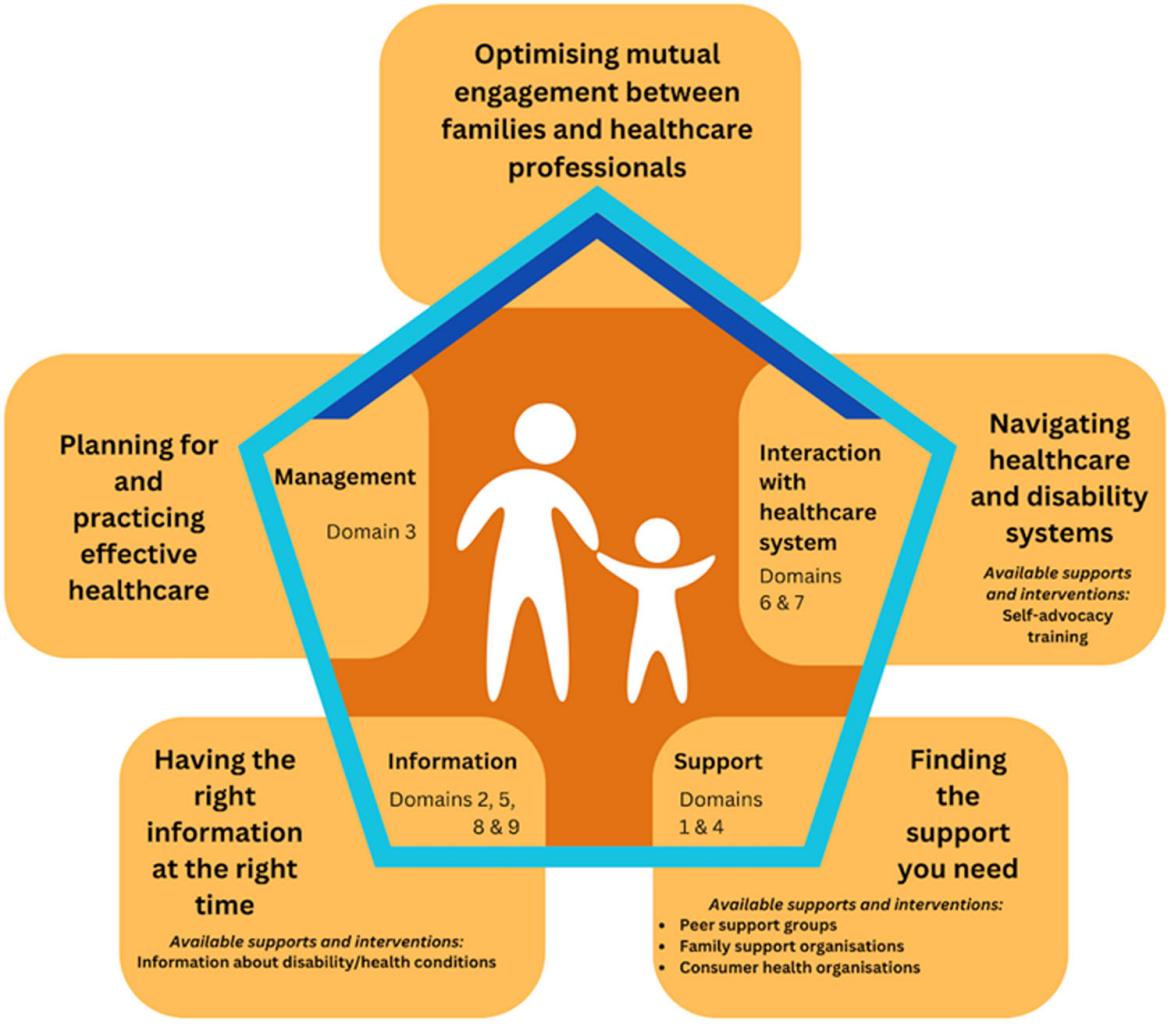
Families' experiences of the healthcare journey and the relationships between the themes and health literacy domains described by Batterham et al. [23]. *Note:* Family experiences are centred and impacted by the themes and corresponding health literacy domains. The outer boxes present this study's themes and the relevant supports and interventions available. The connection between the themes is represented by the pentagon and the dark blue lines indicate that the domains presented for the themes ‘Planning and practising effective healthcare’ and ‘Navigating healthcare and disability support systems’ also mapped to the theme ‘Optimising mutual engagement between families and healthcare professionals’. Related health literacy domains outlined in Batterham et al. [23] are connected to corresponding themes inside the pentagon.

Figure 1 demonstrates how this study's themes map to the health literacy domains outlined by Batterham et al. [23]. The five themes we identified (information outside the hexagon) link to different health literacy domains (information inside the pentagon) and are all connected as represented by the hexagon. Additionally, corresponding relevant supports and interventions are presented for each of the themes and their corresponding health literacy domains. The ability to access information and receive support are foundational aspects of health literacy [24] and are presented at the base of the shape to demonstrate how they scaffold the other domains. These factors enabled parents to effectively oversee their children's health and navigate health services. This aligns with previous research, where parents of children with disability have highlighted the significance of seeking information about their children's health issues and connecting with peer support [25, 26]. Optimising engagement between healthcare providers and parents appears particularly important and so is presented at the top of the pentagon. The corresponding health literacy domains that relate to this theme are consistent with those for Theme 2 and Theme 5. Parents identified how healthcare providers could be vital sources of support and emphasised the significance of feeling heard and backed by healthcare professionals. Qualities of supportive healthcare professionals, including empathy and recognition of parent's perspectives, align with traits of effective healthcare professionals identified by parents in other studies [10, 17]. Similarly, participants' descriptions of how they developed strong advocacy skills align with findings from the wider literature on parental advocacy in healthcare settings [27]. These findings indicate that some dimensions of health literacy (e.g., ability to find good health information; and feeling understood and supported by healthcare professionals) are important for parents of people with different disabilities and health conditions.

In addition to enhancing health information acquisition and utilisation skills, participants indicated that it was necessary to develop specific knowledge and understanding of their child to address their unique challenges. Participants emphasised the need to have a deep understanding of their children, including being able to recognise subtle signs of pain or changes in their health. This was especially pertinent for parents of children with complex health conditions and genetic disorders. These parents sometimes faced the additional challenge of their child's condition being so rare that there was limited tailored information available for them and not a clear path forward to access support. The necessity of developing individualised knowledge of children with complex care needs is not featured in current models of health literacy. This suggests that there are additional and unique dimensions of health literacy that apply to parents of children with intellectual disability and complex health needs.

The synchronisation of this study's findings and the domains of health literacy demonstrate the potential utility of enhancing health literacy skills to effectively aid parents in better managing their children's health conditions. Developing these skills during the early stages of children's development could be critical in reducing the long‐term impact of comorbidities associated with intellectual disability. Similarly, interventions to improve parents' ability to advocate for services for their child can be effective, particularly for those with lower baseline perceived advocacy skills [28]. Increasing in number, disability and carer peer support groups present an opportunity to learn from the experiences of others and have been shown to reduce feelings of isolation and empower parents [29]. Although there is support and interventions addressing advocacy, information, and social support, there appear to be few interventions that support a holistic set of skills, are individualised and enable proactive management of the health of children with disability.

While health literacy supports for parents of children with varied disabilities and chronic conditions have been evaluated, there has been limited focus on the health literacy needs of parents of children with intellectual disability [30, 31]. This study's findings highlight the importance of research to further examine how we can better support parents to develop key health literacy knowledge and skills, targeting their specific health literacy gaps while utilising their strengths. Our data suggest an avenue for effective intervention could be pairing generic information related to comprehensive health literacy skills, with individualised attention to the knowledge and skills required by the family at the time, tailored to their child's specific health needs.

Whilst there was diversity in the healthcare needs of the children, there was less parent‐participant diversity, where the sample predominantly included mothers from metropolitan areas. Analyses did not explore the impacts of intersectional identities (e.g., socioeconomic factors, ethnicity, gender effects on parents' experiences). These factors were not a focus of this study but may powerfully influence how parents navigate healthcare systems and future research should explore these impacts.

### 4.1 Implications for Practice and Policy

This study has implications for policy, service planning, and procurement/funding in the health, disability, and community services sectors. Recognising that families with children with intellectual disability are often navigating between services across these sectors, there is a need to provide clear pathways for families so that access is more equitable and not reliant on the parent's health literacy skills. The vision of equitable access to healthcare training and services for families is critical to improving health and quality of life outcomes across the community [32]. Further, there is a need to consider how families are supported to develop their health literacy skills to optimise health outcomes for their child and who has the responsibility to guide this process.

## Author Contributions

**Thom Nevill:** visualisation, writing–review and editing, writing–original draft, formal analysis, project administration. **Jessica Keely:** conceptualisation, data curation, methodology, investigation, visualisation, writing–original draft, writing–review and editing. **Rachel Skoss:** conceptualisation, writing–review and editing, methodology, writing–original draft, visualisation, formal analysis. **Rachel Collins:** writing–review and editing. **Katherine Langdon:** writing–review and editing. **Jaquie Mills:** writing–review and editing. **Jenny Downs:** writing–review and editing, conceptualisation, investigation, funding acquisition, methodology, visualisation, project administration, supervision.

## Ethics Statement

The study has been approved by the University of Western Australian Human Research Ethics Committee (HREC) – ET000948. All participants provided informed verbal consent.

## Conflicts of Interest

The authors declare no conflicts of interest.

## Supporting information

Supporting information.

## Data Availability

Research data is available upon request subject to ethical approval.
