# Erratum to “What I Wish I Had Known: Examining Parent Accounts of Managing the Health of Their Child With Intellectual Disability”

**DOI:** 10.1111/hex.70178

**Published:** 2025-02-04

T. Nevill, J. Keely, R. Skoss, R. Collins, K. Langdon, J. Mills, and J. Downs, (2025), “What I Wish I Had Known: Examining Parent Accounts of Managing the Health of Their Child With Intellectual Disability,” *Health Expectations* 28, (2025): e70138, https://doi.org/10.1111/hex.70138.

In the list of authors, the surname of second author “Keely” is incorrect. This should have read “Keeley”.

We apologize for this error.

