# Supplementary material for: What I Wish I Had Known: Examining Parent Accounts of Managing the Health of Their Child With Intellectual Disability
Source: Health Expect. 2025 Jan 9;28(1):e70138. doi: 10.1111/hex.70138 (PMC11713037; doi:10.1111/hex.70138)
Supplement: Supplementary file 1 — Supporting information. [file HEX-28-e70138-s001.docx]

Appendix A Interview guides

**Communication, Behaviour, Mental Health, and Sleep Interview Guide**

1. First, I’d like us to talk about communication about health – it could be verbal, or non-verbal.

Prompts

- How does your child communicate when they are not feeling well, or are in pain?
- What has been the ‘aha’ moment for you about recognising they’ve got a health problem?
- What is the most important thing for other families to know?
- What are the most important things that health professionals should know about how your child communicates about their health?

1. If we were to think about the mental health of your child…

Prompts

- What have been the challenges in managing your child’s mental health or mental wellbeing?
- Who has been helpful in supporting your child’s mental wellbeing/mental health?
- What advice have you been given, good or otherwise…
- What has been the most useful advice, or the best thing you’ve done that has supported your child’s mental health?
- What do you wish you’d known earlier about your child’s mental health?
- What is the most important thing you can share to other families?

1. Let’s talk about whether you’ve had any challenges with your child’s sleep. There are several issues that can occur with sleep, for example, it could be from insomnia, with problems either getting to sleep or staying asleep, breathing-related problems like snoring or sleep apnoea, or extreme sleepiness.

Prompts

- What have been the challenges in managing your child’s sleep patterns?
- What is the best advice you’ve received about your child’s sleep, and who gave it?
- What do you wish you’d known earlier about managing your child’s sleep?
- If your child has poor sleep patterns, what else do you think you need to support improvement?
- What do you wish health professionals knew about your child’s sleep and it’s impacts on other aspects of their health?
- What do you think other families need to know?

1. It’s time to talk about behaviour. First, we are going to talk about behaviours that may be considered challenging.

Prompts

- How have you and your child been supported with respect to challenging behaviour?
- What has been the most useful advice that you’ve received? (e.g., Positive behaviour support plan?)
- What have you been told that didn’t work for your child?
- What other supports or advice do you think would be helpful for you?
- What do you wish you’d known earlier?
- What do you think other families need to know?
- What other areas of behaviour do you think are important to talk about?

1. Is there anything else you think is important for us to discuss today?

**Dental Health Interview Guide**

Today we are going to talk about dental care. We are going to talk about three different aspects of care.

1. First, we are going to talk about the preventative care we do at home. So this could include brushing teeth, flossing, and considering the foods that are eaten.

Prompts

- How were you supported in the preventative side of dental care?
- Where did that support, or information come from?
- What do you wish you’d known about preventative dental care?
- What do you think is important for other families to know?

1. Let’s now talk about the interactions with the dentist – so this could be regular dental check-ups, but also the other things that can happen in a typical non-specialist dental clinic.

Prompts

- How did your child access dental care?
- What was helpful in supporting access to dental care?
- What do you wish you’d known?
- What do you think is important for dentists to know about providing services to people with intellectual disability?
- What do you think other families need to know?

1. Sometimes, people will receive dental services in a hospital clinic. This can happen if there is a need for a general anaesthetic as the person with disability may not otherwise be able to tolerate the dental work.

Prompts

- What was most helpful for you/your child if there was a need for dental work to be conducted in hospital?
- What do you wish you’d known?
- What do you think the dentist need to know if they are providing dental care in this situation?
- What do you think other health professionals need to know, in order to better support you/your child?
- What do you think other families need to know if this happens to them?

1. Is there anything else you think is important for us to discuss today?

**Epilepsy Interview guide**

- When your child’s seizures were being investigated and diagnosed, what support or advice did you find most helpful?
- Thinking about the ongoing management of the epilepsy, what support or advice did/do you find most helpful
- How have health professionals explained to you how epilepsy can change at particular periods in life, and what you need to look for?
- What has been the most important thing you’ve learned about the day-to-day management of the condition?
- What health professionals are involved in the care? What do you wish they knew about your child and family?
- What do you wish you’d known before seeking a diagnosis?
- What would you like other families like you to know about epilepsy?
- Is there anything else you think is important for us to discuss today?

**GI Interview Guide**

1. I’d like to start by talking about eating and health. There can be different challenges – for some, the challenge is in limiting food intake, while others it may be getting them to eat enough, or to eat a variety of foods. I’m curious about your experiences in managing your child’s diet.

Prompts

- What challenges have you experienced?
- What advice has been the most helpful for you?
- Who gave it?
- What sort of things were you told that just haven’t worked for your child?
- What do you think is important for other families to know?

1. Let’s talk about swallowing now. Sometimes it can be difficult to identify that a person has a problem with swallowing or know who to talk to about it.

Prompts

- If your child has experienced issues with swallowing, how did you recognise it?
- Who did you seek help from about it?
- How old was the child?
- What do you wish you’d known about the issue?
- How have you changed things – in terms of food that your child eats, how they take medication, and so on?

1. Reflux occurs when the stomach acid irritates the lining of the oesophagus. It can be painful, especially after eating – and gets worse when you lie down. I’m interested in knowing what has worked to manage it.

Prompts

- How did you find out that reflux was an issue?
- What was the most useful advice you were given?
- Who gave it?
- What things have you had to be aware of in managing your child’s reflux?
- What are the most important things for other families to know?

1. Constipation is common and occurs when the waste moves too slowly through the digestive tract or can’t be eliminated effectively. There are many possible causes of chronic constipation. It can be difficult to know when someone is constipated, especially as toileting often happens out of sight.

Prompts

- What are the signs you see when your child is constipated? (e.g. straining, aversion to go to the toilet, behavioural, etc.)
- Where/who have you sought help from to manage the constipation?
- What have you found works best in managing the constipation?
- What are the most important things for other families to know?

1. Is there anything else you think is important for us to discuss today?

**Movement Interview Guide**

1. I’d like to start by talking about your experiences in getting suitable equipment (e.g., wheelchairs, commode, standing frame)

Prompts

- What was your experience in getting a wheelchair that fits?
- What were the impacts of that on your child’s health?
- What were the most important things you’ve learned about your child’s posture?
- Were there any ‘aha’ moments you had that improved the child’s experience?
- What do you wish you’d known earlier?
- What do you think is important for other families to know?
- What about health professionals – what do they need to know?

1. We know that when people are sitting for long periods of time, they can develop issues which may turn into bigger health problems like pressure sores, postural problems, and so on…

Prompts

- What types of things were you told to do to manage these potential issues?
- How did the health professionals talk about the risk of long-term sitting?
- Were you encouraged to give your child floor time or up time?
- How did that work?
- What was the best advice you were given about managing this aspect of your child’s health?
- Who gave that advice?

1. What other useful advice did you receive about your child’s mobility?

Prompts

- What about types of exercise or leisure activities your child can do?
- What advice have you been given about helping your child move for health benefits?
- How did you find balancing food intake and activity levels? Was that ev
- What do you think is most important for other families to know?

1. What do you think other parents need to know about movement?
2. Is there anything else you think is important for us to discuss today?

**Respiratory Health Interview Guide**

1. First, let’s talk about what has been helpful in managing the seasonal viruses and infections that occur. I’m interested in knowing about how it worked to get help mainly from a GP to manage it, as well as when hospitalisation was required.

Prompts

- What support or advice did you get from a GP that you found helpful in managing the virus/infection?
- Where else did you find helpful information from?
- What was that information?
- If/when your child needed hospitalisation, what else do you feel you needed to know?
- If there was a need for ongoing management of the condition, which health professionals were involved?
- How did that get organised?
- What do you wish you’d known earlier about managing the virus/infection?
- What do you think is most important for other families to know?

1. (If participants have had experience with pneumonia, persistent bronchitis etc.)

Prompts

- When you were seeking a diagnosis of some sort for your child, what support or advice did you find most helpful for you, or for your child?
- If you needed follow-up with respiratory specialists, how did you find that experience?
- What do you wish you’d known earlier?
- What do you wish health professionals had known?
- What do you think is the most important thing for other families to know?

1. Now let’s talk about managing other chronic conditions. These could include conditions like asthma or cystic fibrosis, but also when there is a respiratory problem that impacts sleep like snoring or sleep apnoea which may need treatment.

Prompts

- What have you found most helpful in managing these health conditions?
- What supports would have been useful (that you didn’t receive)?
- What do you wish you’d known earlier?
- What do you think is the most important thing for other families to know?

1. Finally, we’ve apparently made it through to the other side of the Covid pandemic. I’m interested to hear your opinions on what worked well for you or where you could have been better supported.

Prompts

- What were the gaps in information (if any)?
- What do you wish health professionals/policymakers knew about intellectual disability?
- In retrospect, what would you have done differently (or wanted to be done differently)?

1. Is there anything else you think is important for us to discuss today?
